# Structural Characterization of an Acidic Polysaccharide from Walnut Green Husks and Its Therapeutic Potential in DSS-Induced Ulcerative Colitis

**DOI:** 10.3390/nu18091351

**Published:** 2026-04-24

**Authors:** Sanawar Mansur, Xin Hu, Xinyu Song, Nuerbiye Jueraiti, Anargvl Mahmut, Fuxiang Luo, Aytursun Abuduwaili, Weihao Wang, Zulfiye Talat, Xieraili Tuerxun

**Affiliations:** 1 College of Chemistry and Chemical Engineering, Xinjiang Agricultural University, Urumqi 830052, China; sanam0405@163.com (S.M.); tianhu230@163.com (X.H.); 18160205140@163.com (X.S.); ayituxunabuduwaili14@mails.ucas.ac.cn (A.A.); 2 College of Uyghur Medicine, Xinjiang Medical University, Urumqi 830017, China; nice1152024@163.com; 3 Key Laboratory of Xinjiang Uyghur Medicine Prescription, Xinjiang Institute of Materia Medica, Urumqi 830011, China; 18999966874@163.com (A.M.); 18299105850@163.com (F.L.); 4 Institute of Chinese Materia Medica, China Academy of Chinese Medical Sciences, Beijing 100700, China; whwang@icmm.ac.cn

**Keywords:** walnut green husk polysaccharide, structure identification, ulcerative colitis, intestinal barrier

## Abstract

**Background/Objectives**: The worldwide occurrence of ulcerative colitis (UC) is increasing, but existing treatments frequently suffer from limited effectiveness and notable side effects. walnut green husk polysaccharide (WGHP) has been shown to exhibit anti-inflammatory and immunomodulatory activities; however, its specific potential and mechanisms of action against colitis remain unclear. This study aimed to evaluate the effectiveness of purified WGHP on (dextran sulfate sodium) DSS-induced UC and elucidate the underlying mechanisms. **Methods**: WGHP-2-2, a primary acidic polysaccharide fraction, was extracted from crude WGHP and analyzed through chromatography and spectroscopy. The therapeutic efficacy of WGHP-2 was assessed using a murine model of DSS-induced UC. Assessments included disease severity (DAI, colon length, histopathology), inflammatory markers (tissue IL-6, TNF-α, IL-10), and intestinal barrier integrity (Claudin-5, Occludin, ZO-1). **Results**: WGHP-2-2 is an acidic polysaccharide with a molecular weight of 15.29 kDa. Its composition includes glucosamine, rhamnose, glucuronic acid, galacturonic acid, glucose, galactose, and arabinose, with respective molar ratios of 0.55, 8.48, 3.06, 65.99, 4.49, 10.86, and 6.57. Methylation and NMR analyses revealed a backbone mainly composed of →4)-α-D-GalpA-(1→ and →2)-α-D-Rhap-(1→ linkages, with side chains or terminal residues such as T-Rhap, T-Galp, T-Glcp, and T-Araf. In vivo, WGHP-2 significantly mitigated DSS-induced UC symptoms in a dose-dependent manner. Specifically, the high-dose group (123 mg/kg) markedly attenuated colon shortening and improved histological architecture, including the restoration of colonic crypts. WGHP-2 effectively reduced pro-inflammatory cytokines IL-6 and TNF-α in colon tissues, while increasing the anti-inflammatory cytokine IL-10. **Conclusions**: WGHP-2 mitigates DSS-induced UC by inhibiting pro-inflammatory cytokines (IL-6, TNF-α), increasing IL-10 levels, and improving intestinal barrier integrity through the upregulation of tight junction proteins. These results position WGHP-2 as a promising lead compound for developing functional foods for UC.

## 1. Introduction

Ulcerative colitis (UC) is a chronic, idiopathic inflammatory bowel disease characterized by relapsing colonic mucosal inflammation, manifesting as diarrhea, hematochezia, and abdominal pain [1,2,3]. With rising global incidence, UC imposes a substantial economic burden and significantly impairs patients’ psychological well-being and social functioning, while increasing colorectal cancer risk [4,5]. Although its etiology involves complex genetic, environmental, and immune interactions [6], its pathogenesis is centrally driven by intestinal barrier dysfunction. In UC, goblet cell-derived MUC2 depletion thins the protective mucus layer, while the downregulation of tight junction (TJ) proteins (e.g., Occludin, Claudin-1, and ZO proteins) increases epithelial permeability [7,8,9,10]. This compromised barrier facilitates bacterial and antigen translocation into the submucosa, triggering aberrant immune responses and sustained inflammation.

The current therapeutic strategies for UC primarily rely on non-specific anti-inflammatory agents, targeted biologics, and Janus kinase (JAK) inhibitors [11,12]. However, the clinical utility of these small-molecule drugs is often limited by significant adverse effects and variable efficacy, underscoring an urgent need for novel therapeutics with improved safety profiles. Consequently, the exploration of bioactive compounds derived from natural products has emerged as a promising avenue for UC management [13].

Among these, pectic polysaccharides have demonstrated substantial therapeutic potential in experimental colitis by targeting two key pathological features of UC: (i) attenuating excessive inflammatory responses through the suppression of pro-inflammatory mediators; and (ii) enhancing intestinal barrier integrity by upregulating the expression of TJ proteins such as ZO-1 and Occludin [14,15,16].

*Juglans regia* L. (walnut), a renowned medicinal and edible resource, is rich in diverse bioactive constituents. While existing research has predominantly focused on the kernel, other walnut by-products—such as the green husk, hard shell, septum (inner partition), and leaves—remain underutilized despite their high content of anti-inflammatory compounds. Preliminary studies indicate that walnut septum extracts possess potent anti-UC activity [17]. Similarly, walnut green husks, traditionally used for heat and detoxification, have been shown to possess notable anti-inflammatory and colon-protective properties [18,19]. Furthermore, polysaccharides extracted from walnut husks have exhibited significant hepatoprotective effects against liver inflammation [20]; however, their specific efficacy and underlying mechanisms in the context of UC remain unexplored.

Currently, the disposal of walnut husks as agricultural waste contributes significantly to environmental pollution. Therefore, the extraction and valorization of bioactive polysaccharides from walnut husks represent a dual-benefit strategy: it offers a sustainable source of lead compound while addressing environmental concerns through waste reduction [21]. In this study, we aimed to isolate WGHP-2-2, a highly purified homogeneous fraction derived from walnut green husk, and to elucidate its structure using multidimensional spectroscopy. Furthermore, we evaluated the therapeutic potential of the active fraction WGHP-2 in a DSS-induced ulcerative colitis model using BALB/c mice. DSS disrupts the colonic epithelial barrier by damaging surface epithelial cells and degrading the mucus layer, leading to bacterial translocation, activation of innate immunity, and excessive production of pro-inflammatory cytokines such as TNF-α and IL-6—pathological features that closely resemble those of human ulcerative colitis. Our results demonstrate that WGHP-2 exerts significant therapeutic effects by alleviating colon shortening and suppressing TNF-α and IL-6 expression. Furthermore, the study investigated the mechanisms underlying its restoration of intestinal mucosal barrier function. We anticipate that these findings identify WGHP-2 as a promising lead compound for developing functional foods, thereby expanding the structural and activity profile of walnut green husk acidic polysaccharides.

## 2. Materials and Methods

### 2.1. Materials and Reagents

The walnut green husk, sourced from Aksu in the Xinjiang Uygur Autonomous Region of China, was authenticated by Associate Professor Shengjun Ma from the College of Food Science and Pharmacy at Xinjiang Agricultural University. DEAE 650M was sourced from Tosoh Company (Tokyo, Japan), and Sephacryl-S200 from Cytiva Inc., (Marlborough, MA, USA). Dextran sulfate sodium salt (DSS) and dialysis membranes were acquired from Beijing Solarbio Technology Co., Ltd. (Beijing, China). An ELISA kit was obtained from Shanghai Jianglai Industrial Co., Ltd. (Shanghai, China), while sulfasalazine (SASP) tablets were purchased from Shanghai Xinyi Tianping Pharmaceutical Co., Ltd. (Shanghai, China). Hematoxylin and eosin (HE) staining solution, neutral gum, EDTA antigen repair solution, PBS buffer, erythrocyte lysis buffer, Claudin-5, Occludin, and ZO-1 were all sourced from Wuhan Xavier Technology Co., Ltd. (Wuhan, China). The remaining chemical reagents used were of analytical grade purity.

### 2.2. Preparation of Polysaccharide from Walnut Green Husk

#### 2.2.1. Polysaccharide Extraction

The preparation of WGHP began with pulverizing dried walnut husks and sieving them (60 mesh). Defatting and decolorization were achieved by treating the powder with petroleum ether and absolute ethanol (5:1 mL/g). Hot water extraction of the residue was performed at 90 °C for 2 h (solid:liquid = 1:20 g/mL). The cooled extract was filtered, reduced in volume by two-thirds, and centrifuged (4000 rpm, 10 min). Dialysis of the supernatant was carried out for 48 h (8–10 kDa membrane). Crude polysaccharides were isolated by adding four volumes of absolute ethanol to the dialysate and incubating at 4 °C for 12 h. The resulting pellet was collected via centrifugation (6000 rpm, 10 min), dissolved in water, and lyophilized to yield WGHP.

#### 2.2.2. Polysaccharide Isolation

The crude WGHP (10 mg/mL) underwent decolorization and desalting on an AB-8 macroporous resin. Subsequently, the purified solution was applied to a DEAE-650M anion-exchange column (50 mm × 500 mm) and eluted with a 0–0.5 mol/L NaCl gradient (2 mL/min). Monitoring via the anthrone–sulfuric acid reaction revealed three main fractions: neutral WGHP-1 and acidic WGHP-2 and WGHP-3. Following concentration, dialysis (3500 Da MWCO), and lyophilization, the most abundant fraction, WGHP-2, was further resolved on a Sephacryl S-200 gel filtration column (16 mm × 750 mm). Elution with distilled water at 0.5 mL/min produced two sub-fractions, WGHP-2-1 and WGHP-2-2.

### 2.3. Structural Confirmation of Polysaccharide

#### 2.3.1. UV-Vis Spectrum

The polysaccharide was dissolved in deionized water (0.1 mg/mL) for UV-Vis spectroscopy. Spectral data were collected from 200 to 800 nm, referencing deionized water as the blank.

#### 2.3.2. FT-IR Spectra

The polysaccharide powder was directly applied to the ATR crystal, and spectra were recorded over a wavenumber range of 4000 to 400 cm^−1^. FT-IR spectra were obtained with a Shimadzu IRTracer-100 spectrometer (Shimadzu, Kyoto, Japan) using an ATR accessory.

#### 2.3.3. Determination of Molecular Weight

Accurately weigh 5 mg of the sample, dissolve it in 0.05 M NaCl solution to prepare a test solution with a concentration of 5 mg/mL, filter the solution, and then subject it to HPGPC analysis. The HPGPC system comprised a Waters 1515 pump and a Waters 2410 RI detector (Waters Corporation, Milford, MA, USA). For chromatographic separation, three Shodex Ohpak aqueous SEC columns (SB-803 HQ, SB-804 HQ, SB-805 HQ; 8 mm × 300 mm; Nippon Shokubai, Osaka, Japan) were connected in series. The operating parameters included an isocratic mobile phase of 0.05 M NaCl, a flow rate of 0.65 mL/min, and a column temperature of 40 °C. Each analysis involved an injection volume of 30 µL.

#### 2.3.4. Determination of Monosaccharide Composition

Refer to Huang’s method [22]. WGHP-2-2 (5 mg) underwent hydrolysis using 1 mL of 2 mol/L trifluoroacetic acid (TFA) at 120 °C for 2 h. The resulting hydrolysates were subsequently dried, and any remaining TFA was eliminated through co-evaporation with methanol (3 mL), repeated 2–3 times. The dried residues were then redissolved in 5 mL of distilled water. Hydrolysate (0.2 mL) was derivatized by reacting with equal volumes of 0.5 mol/L NaOH and 0.5 mol/L 1-phenyl-3-methyl-5-pyrazolone (PMP) at 70 °C for 1 h. Following cooling and neutralization with 0.2 mL of 0.5 mol/L HCl, the mixture underwent triple extraction with 1 mL of chloroform to strip away residual PMP. The aqueous layer was collected, and a 0.3 mL aliquot was diluted to 1 mL with deionized water for subsequent HPLC analysis.

Separation was conducted on a Thermo U3000 HPLC system (Thermo Scientific, Waltham, MA, USA) using a ZORBAX Eclipse XDB-C18 column (4.6 mm × 250 mm, 5 µm; Agilent Technologies, Santa Clara, CA, USA). The mobile phase comprised acetonitrile/0.1 mol/L phosphate buffer (17:83, *v*/*v*) pumped at 0.8 mL/min. Operating conditions included a column temperature of 30 °C, UV detection at 250 nm, and a 10 µL injection volume.

#### 2.3.5. Scanning Electron Microscopy (SEM) Analysis

Polysaccharide samples were sputter-coated with a thin layer of gold and mounted onto an aluminum stub. The surface morphology was then examined using SEM, with images captured at various optimized magnifications.

#### 2.3.6. Methylation Analysis

Refer to Zhang’s method [23]. To reduce uronic acids, polysaccharide samples (10 mg in 1 mL water) underwent carbodiimide-mediated activation with 1 mL of 100 mg/mL EDC for 2 h. The reaction was then quenched and reduced by adding 1 mL of 2 M imidazole and 1 mL of 30 mg/mL NaBD_4_, with further incubation for 3 h. The process was stopped with 100 µL acetic acid, and the product was purified via dialysis (24 h) and lyophilization. Methylation was conducted by dissolving 1 mg of the reduced sample in 500 µL anhydrous DMSO, reacting with 1 mg fine NaOH powder for 30 min, and finally treating with 50 µL methyl iodide for 1 h. The product was extracted into dichloromethane (DCM), washed three times with water, and evaporated. The polymer was hydrolyzed (100 µL of 2 M TFA, 121 °C, 90 min), and the hydrolysate was reduced with 50 µL each of 2 M NH_3_ and 1 M NaBD_4_ at room temperature for 2.5 h. The resulting product was then acetylated with 250 µL of acetic anhydride at 100 °C for 2.5 h. Post water quenching, the mixture was extracted into DCM. The final organic layer was collected for analysis. The GC-MS system comprised an Agilent 7890A unit interfaced with a 5977B quadrupole MS, featuring an HP-5MS column (30 m × 0.25 mm × 0.25 µm; Agilent, Santa Clara, CA, USA). With helium as the carrier gas (1.0 mL/min), 1 µL aliquots were introduced via a split injector (10:1 ratio) at 260 °C, incorporating a 2.2 min solvent delay. The thermal gradient started at 50 °C (1 min), ascended to 130 °C at 50°C/min, then proceeded to 230 °C at 3 °C/min, concluding with a 2 min isothermal hold.

Electron ionization (70 eV) was employed to generate mass spectra in full-scan mode (*m*/*z* 30–600), with the ion source and quadrupole temperatures maintained at 230 °C and 150 °C, respectively.

#### 2.3.7. NMR Detection of Structure Elucidation

NMR spectra (^1^H, ^13^C, COSY, HSQC, HMBC, and NOESY) of WGHP-2-2, dissolved in D_2_O at 10 mg/mL, were obtained on an Agilent VNMRS 600, (Agilent, Santa Clara, CA, USA).

### 2.4. Improvement of Ulcerative Colitis by WGHP in Mice

**Figure 1:**
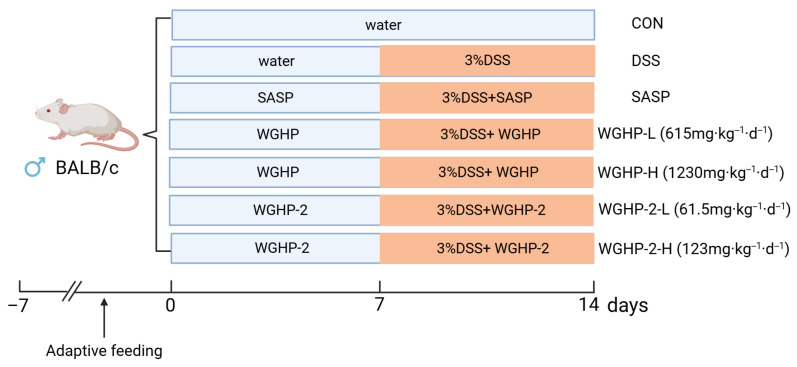
Technical roadmap for experimental DSS-induced acute ulcerative colitis in mice.

Eight-week-old male BALB/c mice (20 ± 2 g), obtained from Xinjiang Medical University’s Laboratory Animal Management Center (Urumqi, China), were kept in a specific pathogen-free (SPF) facility maintained at 22–24 °C and 50–60% humidity, with free access to food and water. All experimental procedures followed the ethical standards set by the Animal Ethics Committee (Approval No. IACUC-JT-20250527-47). A total of 70 mice were randomly divided into 7 groups, with 10 mice in each group: the control group, DSS group, low-dose WGHP group (WGHP-L group; 615 mg/kg), high-dose WGHP group (WGHP-H group; 1230 mg/kg), low-dose WGHP-2 group (WGHP-2-L group; 61.5 mg/kg), high-dose WGHP-2 group (WGHP-2-H group; 123 mg/kg), and SASP group (positive control; 250 mg/kg)—a first-line ulcerative colitis drug metabolized in the colon to 5-aminosalicylic acid. Dosing regimens were derived from the Henan Province TCM Decoction Pieces Standard for Qinglongyi [24]. The protocol specifies a daily dosage range of 15–30 g, which was further adjusted based on the extraction yields of WGHP (approximately 20%, *w*/*w*) and WGHP-2 (approximately 2%, *w*/*w*), as well as a species-specific conversion factor (12.3). UC was established by administering 3% (*w*/*v*) DSS in drinking water ad libitum for 7 days to the experimental groups; the control cohort received distilled water. The experiment was concluded on day 8. All mice were deeply anesthetized via intraperitoneal injection of pentobarbital sodium (100 mg/kg). Once unconsciousness was confirmed, blood was collected via retro-orbital venous plexus puncture, followed by exsanguination to ensure humane euthanasia. The experimental flowchart is shown in Figure 1.

#### 2.4.1. Observation of Mouse Symptoms and Specimen Collection

Daily body weight measurements were taken at a fixed time. The Disease Activity Index (DAI) comprised cumulative scores (0–4) for weight loss, stool consistency, and rectal bleeding. Weight loss thresholds were set at 0% (score 0), 1–5% (1), 5–10% (2), 10–20% (3), and >20% (4). Stool consistency ranged from normal (0) to diarrhea (4), including slightly soft (1), soft (2), and semi-liquid (3) stages. Rectal bleeding, assessed by visual inspection, was scored from 0 (negative) to 4 (severe/gross bleeding). Following euthanasia, colons were harvested and their lengths recorded. The tissues were then segmented into two portions: one fixed in 4% paraformaldehyde for histopathological examination, and the other snap-frozen and stored at −80 °C for cytokine analysis.

#### 2.4.2. Analysis of Inflammatory Factors in UC Mice

Inflammatory factor levels in mouse serum and tissues were measured using specific ELISA kits.

#### 2.4.3. H&E Staining and Histopathological Analysis

Following fixation in 4% paraformaldehyde, 1.0 cm distal colon samples were processed via graded ethanol dehydration, xylene clearing, and paraffin embedding. Resulting 4 µm sections were then prepared and underwent hematoxylin and eosin (H&E) staining for histopathological assessment.

#### 2.4.4. Colonic Immunohistochemical Analysis

Immunohistochemistry (IHC) was conducted on paraffin-embedded colon sections, prepared according to Section 2.4.3, utilizing a universal IHC kit. The sections were deparaffinized, rehydrated, and washed three times with PBS. Antigen retrieval was performed via heat-induced epitope retrieval (HIER) by microwaving the sections in citrate buffer (pH 6.0) at 100 °C for 8 min, followed by a 30 min cooling period at room temperature. Endogenous peroxidase activity was subsequently quenched, and non-specific binding sites were blocked by incubating the sections with 3% hydrogen peroxide and normal goat serum, respectively, for 20 min each in a humidified chamber. Sections were probed with primary antibodies targeting ZO-1, Occludin, and Claudin-5 (each diluted 1:400) and incubated overnight at 4 °C. Following PBS washing, a biotin-conjugated secondary antibody and streptavidin–horseradish peroxidase were applied in succession, with each step lasting 20 min at room temperature. Peroxidase activity was visualized by exposing sections to 3,3′-diaminobenzidine (DAB) for ~20 s, after which the reaction was quenched using distilled water. Following a 1 min hematoxylin counterstain, sections were differentiated with 1% acid alcohol and blued in tap water (10 min). The slides were then dehydrated in graded ethanol, cleared with xylene, and coverslipped with neutral balsam. Positive protein expression was confirmed under a light microscope.

### 2.5. Statistical Analysis

Data analysis was conducted in SPSS v25.0 (IBM Corp., Armonk, NY, USA), and figure preparation was conducted in GraphPad Prism 10.1 (GraphPad Software, San Diego, CA, USA). Continuous data were analyzed using one-way ANOVA with Tukey’s multiple comparisons test. Results are expressed as mean ± SD, where *n* represents the number of biologically independent samples. Statistical significance is indicated as * *p* < 0.05, ** *p* < 0.01, and *** *p* < 0.001.

## 3. Results

### 3.1. Preparation and Purification of Polysaccharides

**Figure 2:**
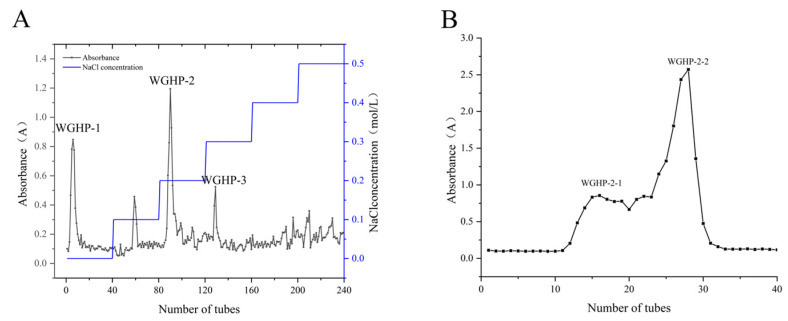
Elution curve of WGHP. Note: (**A**): DEAE-650M elution curve of WGHP; (**B**): Sephacryl S-200 elution curve of WGHP-2-2.

Crude polysaccharides, designated as WGHP, were isolated from walnut husk via hot water extraction followed by ethanol precipitation. DEAE-650M anion exchange resin was employed for separation, followed by stepwise elution using NaCl solutions ranging from 0.1 mol/L to 0.5 mol/L. Combined with the anthrone and sulfuric acid chromogenic method, the elution curve of absorbance changing with the number of elution tubes was drawn (Figure 2A). Elution profiling revealed three separate polysaccharide components, which were isolated and named WGHP-1, WGHP-2, and WGHP-3. The WGHP-2 fraction underwent additional purification in a Sephacryl S-200 column, with its elution monitored using the anthrone–sulfuric acid assay (Figure 2B), ultimately yielding the homogeneous subfraction WGHP-2-2.

### 3.2. UV-Vis Spectroscopic Analysis

**Figure 3:**
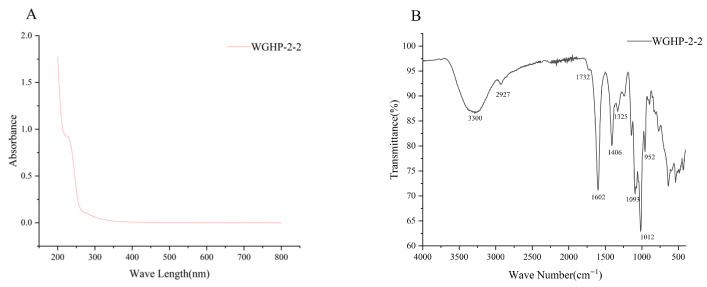
UV-Vis and FT-IR spectra of WGHP-2-2. Note: (**A**): UV-Vis; (**B**): FT-IR.

The UV spectrum of WGHP-2-2 is shown in Figure 3A. The absorbance diminished as the wavelength increased, aligning with typical plant polysaccharide properties. The lack of detectable absorbance at 260 nm, 280 nm, and 520 nm implies that WGHP-2-2 is essentially free of nucleic acids, proteins, and pigments.

### 3.3. FT-IR Spectroscopic Analysis

The FT-IR spectrum of WGHP-2-2 (Figure 3B) displays a strong and broad absorption band around 3300 cm^−1^, corresponding to O–H stretching vibrations; its broadened shape indicates extensive hydrogen bonding. Additionally, a distinct peak at 2927 cm^−1^ is assigned to C–H stretching modes. Notably, the presence of asymmetric (1602 cm^−1^) and symmetric (1406 cm^−1^) carboxylate (–COO^−^) stretching validates the abundant uronic acids (GalA/GlcA) detected previously. Additionally, the pronounced peak at ~1012 cm^−1^, arising from C–O–C glycosidic bonds and pyranose ring C–O stretching, verifies the structural integrity [25].

### 3.4. Structural Characterization

#### 3.4.1. Molecular Weight of Polysaccharides

**Figure 4:**
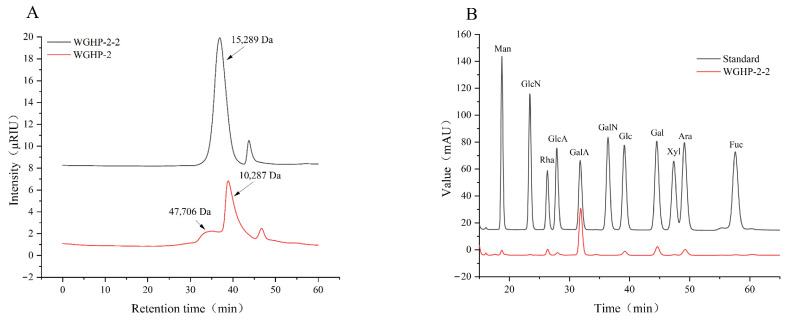
Molecular weight and monosaccharide composition of WGHP-2-2. Note: (**A**): molecular weight; (**B**): composition of monosaccharides.

HPGPC analysis revealed that WGHP-2 comprised two main components with molecular weights of 47.71 kDa and 10.28 kDa. Following additional purification, WGHP-2-2 exhibited a single symmetric peak with a molecular weight of 15.29 kDa, indicating enhanced homogeneity. The elution behavior reflecting molecular size is displayed in Figure 4A, and the quantitative molecular weight parameters for WGHP-2 and WGHP-2-2 are listed in Table 1.

#### 3.4.2. Composition of Monosaccharides

The monosaccharide composition of WGHP-2-2 was determined through HPLC after PMP derivatization. Monosaccharide composition analysis revealed that WGHP-2-2 consists of glucosamine (GlcN), rhamnose (Rha), glucuronic acid (GlcA), galacturonic acid (GalA), glucose (Glc), galactose (Gal), and arabinose (Ara) in molar ratios of 0.549:8.481:3.061:65.991:4.493:10.859:6.566. Among these, galacturonic acid (GalA) was the predominant component, accounting for approximately 66.0% of the total monosaccharides. The results showed that WGHP-2-2 may be a pectic polysaccharide; the results are shown in Figure 4B.

#### 3.4.3. SEM Morphology of Polysaccharides

**Figure 5:**
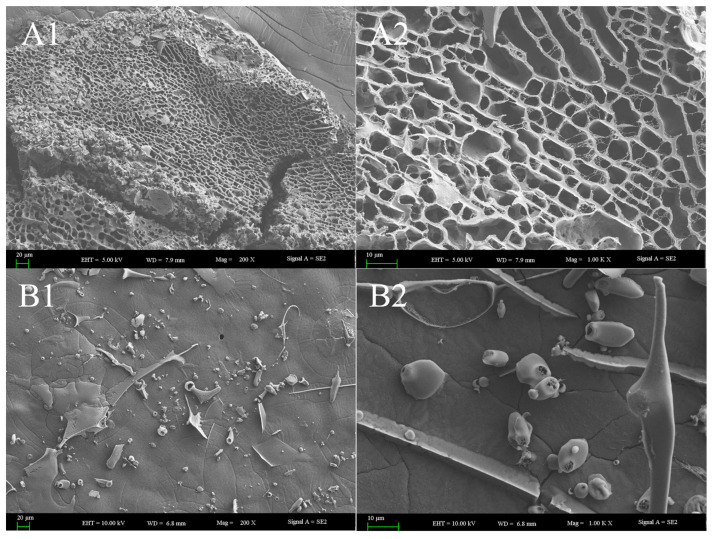
SEM images of WGHP. Note: (**A**): SEM images of WGHP-2; (**B**): SEM images of WGHP-2-2. Magnifications: 1: 200×; 2: 1000×.

The surface morphology and microstructure of the polysaccharides were investigated via scanning electron microscopy (SEM) utilizing a Zeiss Supra55 VP (Carl Zeiss AG, Oberkochen, Germany) instrument. As illustrated in Figure 5(A1,A2), WGHP-2 exhibited a dense, honeycomb-like porous structure characterized by uneven pore sizes and interconnected walls, forming a complex three-dimensional network with irregular contours. This porous architecture, which results in a high specific surface area upon drying, likely facilitates the solubility of the polysaccharide. In contrast, WGHP-2-2 displayed a relatively smooth surface interspersed with irregular network cracks and scattered particulate deposits (Figure 5(B1)). These particles exhibited heterogeneous morphologies, including blocky and needle-like shapes. Under higher magnification, oval and spherical particles, along with rod-like structures resembling fibers or crystals, were clearly visible, and the surface cracking appeared more pronounced. The polysaccharide’s heightened specific surface area and associated structural traits empower it to excel in adsorptive, hydrating, thickening, encapsulating, and reactive roles.

#### 3.4.4. Sugar Residue Studies Based on Methylation Reactions

**Figure 6:**
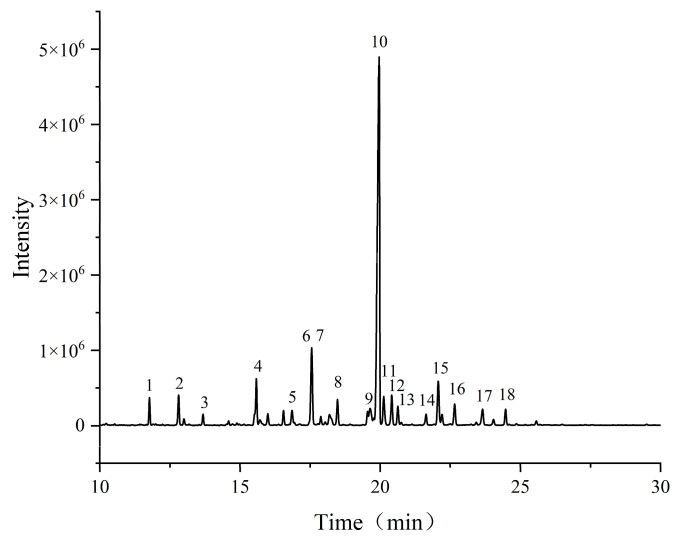
GC–MS total ion chromatogram of the methylated derivatives of WGHP-2-2.

Methylation analysis coupled with GC-MS (Figure 6) identified the following key residues in WGHP-2-2 (Table 2): 1,4-GalpA (54.148%), T-GalpA (5.866%), 1,2-Rhap (5.228%), 1,3,4-GalpA (4.600%), T-Galp (3.489%), and T-Rhap (3.148%). T-Araf, T-Rhap, and T-GalpA constitute the major non-reducing terminal residues, whereas the polysaccharide backbone is built predominantly from alternating →4)-GalpA-(1→ and →1)-Rhap-(2→ linkages. The main junction sites are O-1 and O-2; it also demonstrated that WGHP2-2 was an RG-I pectic polysaccharide. The above structural information is in general agreement with the monosaccharide composition analysis of WGHP-2-2.

#### 3.4.5. NMR-Based Polysaccharide Structure Determination

**Figure 7:**
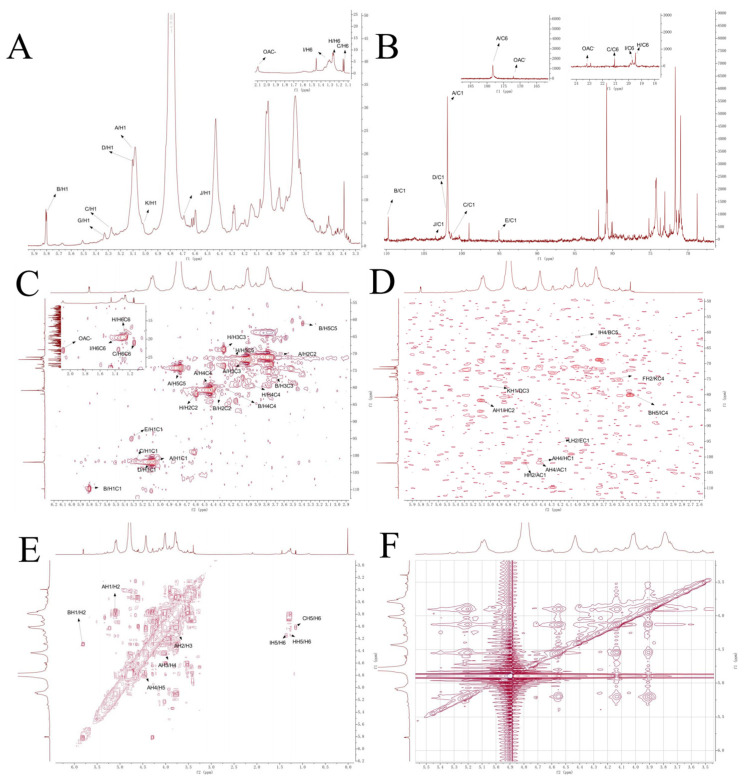
NMR spectrum of WGHP-2-2 in D_2_O. Note: (**A**): nuclear magnetic resonance hydrogen spectrum; (**B**): nuclear magnetic resonance carbon spectrum; (**C**): HSQC; (**D**): HMBC; (**E**): COSY; (**F**): NOESY.

The ^1^H-NMR spectrum of WGHP-2-2 (Figure 7A) showed that the strong peak at δ 4.79 ppm was the solvent peak of D_2_O. The ^1^H NMR spectrum shows extensive overlap in the sugar ring proton region (δ 3.5–4.4 ppm; H2-H6), embedded within a broader envelope (δ 3.4–5.3 ppm). In contrast, the terminal/anomeric region (δ 4.45–5.85 ppm) exhibits multiple resolvable peaks. The terminal hydrogen signals of 1,4-GalpA(A), T-Ara(B), T-Rha(C), 1,3,4-GalpA(D), T-GalpA(F), T-Galp(G), 1,2-Rha(H) and T-Glcp (J) were assigned to δ 5.08, 5.80, 5.28, 5.08, 5.08, 5.32, 5.28 and 4.69 ppm. The end-matrix subsignal concentration appeared around δ 5.11 ppm, indicating that WGHP-2-2 contained an α-configuration sugar loop structure. The signal at δ 1.13 ppm is assigned to the C6-methyl protons of rhamnose residues, while a distinct resonance at δ 2.08 ppm arises from acetyl groups (–OAc), confirming the partial O-acetylation of α-GalpA units [26].

In the ^13^C-NMR spectrum (Figure 7B), WGHP-2-1 showed multiple terminal carbon signals in the δ 90–110 ppm range, mainly distributed around δ 101 ppm. The anomeric proton signals in the ^1^H NMR spectrum, appearing in the characteristic downfield region (δ 4.9–5.8 ppm), indicate that the majority of glycosidic linkages adopt the α-configuration. The signal overlap in the δ 60–82 ppm range was obvious, primarily arising from the ring carbon resonances (C2–C6) of the sugar residues. A signal at δ 178.25 ppm is indicative of a non-esterified uronic acid carboxyl group, whereas the resonance at δ 172.03 ppm arises from the carbonyl carbon of an O-acetyl substituent attached to α-GalpA. The methyl carbon resonances of Rha residues appear at δ 19.41, 19.71, and 21.07 ppm, consistent with C6–CH_3_ environments [27]. The terminal carbonation degree shifts in each residue. 1,4-GalpA(A), T-Ara(B), T-Rha(C), 1,3,4-GalpA(D), T-GalpA(F), T-Galp(G), 1,2-Rha(H) and T-Glcp(J) are respectively δ 101.89, 109.74, 101.28, 101.89, 101.89, 95.07, 101.38 and 103.47 ppm.

Furthermore, the HSQC (Figure 7C) spectrum has the following key C-H correlation signals: δ 5.08/101.89, 5.80/109.74, 5.28/101.28, 5.08/101.89, 5.08/101.89, 5.32/95.07, 5.28/101.38, 4.69/103.47, etc., corresponding to each side of sugar residues C1/H1. The cross peak at δ 2.08/22.91 ppm is attributed to the hydrogen/carbon signal of the acetyl group, indicating that part of α-GalpA residues exists in an acetylated form [28]. The sugar residue T-Araf shows its characteristic H1/C1 signal at δ 5.80/109.74 ppm, combining HSQC with COSY (Figure 7E). The chemical shifts in H2/C2, H3/C3, H4/C4 and H5/C5 were 4.29/80.16, 3.73/74.81, 4.28/80.60 and 3.39/61.14, respectively [29]. Residue C(T-Rha) was confirmed by the H6/C6 signal at δ 1.14 (1.15)/21.07 ppm, and was classified as H5 combined with the relevant signal at δ 4.01 ppm in the COSY spectrum; its C5 chemical shift was further determined to be 71.13 ppm using the HMBC (Figure 7D) spectrum. The corresponding values of H4/C4, H3/C3, H2/C2 and H1/C1 were δ 3.86/73.60, 4.28/68.79, 4.51/81.89 and 5.28/101.38, respectively [30,31].

The sugar residue D(1,3,4-GalpA) was assigned to its H5/C5 by combining HSQC and COSY through C6 as the uronic acid carbonyl carbon signal at δ 178.25 ppm. The chemical shifts in H5/C5, H4/C4, H3/C3, H2/C2 and H1/C1 were δ 4.83/75.18, 4.40/80.86, 4.07/77.14, 3.78/70.99 and 5.11/101.83, respectively [29]. The sugar residue E (1, 3-Galp) was assigned to H2/C2, H3/C3, H4/C4, and H5/C5 by displaying its characteristic H1/C1 signal at δ 5.32/95.07 ppm and combining HSQC with COSY. H6/C6 chemical shifts were δ 3.66/70.89, 3.83/79.00, 3.92/71.23, 3.59/73.21, and 3.50/61.13, respectively. The sugar residue F (T-GalpA) was classified as H5/C5 by combining HSQC and COSY through C6 as the aldehyde carbonyl carbon signal at δ 178.25 ppm. The chemical shifts in H5/C5, H4/C4, H3/C3, H2/C2 and H1/C1 were 4.79/74.22, 4.27/73.68, 4.01/71.69, 3.78/70.99 and 5.08/101.89, respectively [32]. The sugar residue G(T-Galp) was assigned based on its characteristic anomeric signal at δ 5.32/95.07 ppm (H1/C1). By correlating HSQC and COSY spectra (Figure 7D), the remaining proton–carbon pairs were identified as H2/C2 (δ 3.52/70.89), H3/C3 (δ 3.73/67.92), H4/C4 (δ 3.92/71.23), H5/C5 (δ 3.59/73.21), and H6/C6 (δ 3.50/61.13) [33]. In a similar way, the chemical shifts in residue H (1, 2-Rhap) were δ5.28/101.38, 4.59/81.84, 4.28/68.79, 3.92/73.68, 4.14/71.23, and 1.25 (1.26)/19.48 ppm for the residue H (1, 2-Rhap) [34]; δ5.28/101.38, 4.62/81.00, 4.28/68.79, 3.98/80.02, 4.15/70.86, and 1.30 (1.32)/19.72 ppm for the residue I (1,2, 4-Rhap) [35]; δ 4.69/103.47, 3.41/72.49, 3.90/73.37, 3.65/72.25, 3.53/73.22 and 3.44/63.64 ppm for the residue J (T-Glcp) [36]; δ5.02/103.47 ppm aδ3.64/70.75, 3.93/72.36, 3.52/74.83, not specified (ns), and 178.25 for the residue K (1,4-GlcpA) [37]; δ5.08/101.89, 4.20/73.37, 4.43/80.79, ns, and 178.25 ppm for the residue L (1,2,4-GalpA); and δ5.08/101.89, 3.78/70.99, ns, 4.43/80.79, ns, and 172.04 ppm for the residue M (1,4,6-GalpA).

HMBC spectrum analysis revealed a cross-peak at δH/δC 4.79/178.25 ppm, assigned to the correlation between H-5 and the carboxyl carbon (C-6) of residue A, identified as →4)-α-D-GalpA-(1→. This assignment was further supported by HSQC data, which gave ^1^H/^13^C chemical shifts for H2/C2, H3/C3, H4/C4, and H5/C5 as δ 4.15/79.41, 4.15/79.41, 4.40/81.26, 4.97/75.84, and 5.11/73.41, respectively.

Additionally, another HMBC cross-peak at δ 5.17/173.65 ppm was assigned to the H-5/C-6 correlation of an acetylated →4)-α-D-GalpA-(1→ residue. The corresponding HSQC signals for this modified unit were assigned as H2/C2: δ 4.03/79.60, H3/C3: δ 4.14/81.59, H4/C4: δ 4.98/76.03, and H5/C5: δ 5.17/74.45.

Furthermore, the linkage relationships between sugar residues were inferred from HMBC and NOESY spectra. The peak at 4.43/101.89 ppm indicates that the sugar residue A(H4) is associated with A(C1), and A(H1) is observed to be associated with A(H4) in NOESY (Figure 7F), indicating the existence of a →4)-α-GalpA-(1→4)-α-GalpA-(1 linkage mode in WGHP-2-2. The peak at 5.08/81.84 ppm indicates that the sugar residue H(C2) is associated with A(H1), the peak at 4.62/101.89 ppm indicates that the sugar residue H(H2) is associated with A(C1), the peak at 4.43/101.38 ppm indicates that the sugar residue A(H4) is associated with H(C1), and the peak at 5.08/81.84 ppm indicates that the sugar residue H(C2) is associated with A(H1). Moreover, the observation of a H (H1) and A (H4) correlation in NOESY (Figure 7F) indicates the existence of a linkage mode of →4)-α-GalpA-(1→2)-α-Rhap-(1→4)-α-GalpA-(1.

The peak at 5.02/77.14 ppm in HMBC indicates that K (H1) is associated with D (C3), indicating that the 1, 4-GlcpA-residue is linked to →3,4)-α-D-GalpA-(1→ by an O-1 bond, i.e., 4)-α-GlcpA-(1→3,4)-α-D-GalpA-(1→. The peak at 3.52/74.83 ppm indicates that F (H2) is correlated with K (C4) and indicates that the 1)-α-GlcpA-(4→ residue is linked to T-Galp by an O-2 bond, namely 1)-α-GlcpA-(4→T-Galp. For the combined D and K connection, the connection here is 1,4)-α-D-GalpA-(3→1)-α-GlcpA-(4→T-Galp.

**Figure 8:**
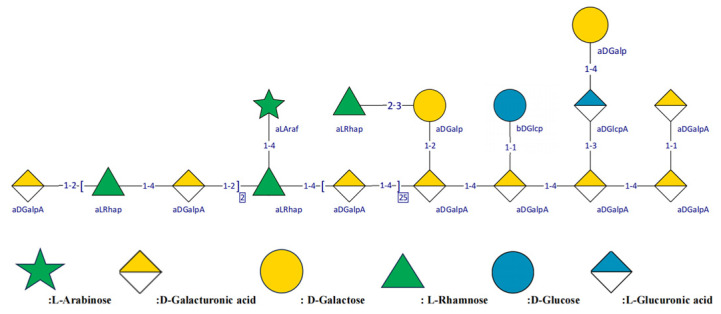
Possible structure of WGHP-2-2.

The peak at 4.20/95.07 ppm in HMBC indicates that L (H2) is related to E (C1) and indicates that the 1, 4)-GalpA-(2→ residue is linked to →1)-α-D-Galp-(3→ by an O-2 bond, namely 3)-α-GalpA-(1→2,4)-β-D-Galp-(1→). The peak at 3.98/61.14 ppm indicates that I (H4) is associated with B (C5), and the peak at 3.39/80.02 ppm indicates that B (H5) is associated with I (C4), indicating that the 1,2)-Rhap-(4→ residue is linked to T-Ara by an O-5 bond, namely 1,2)-Rhap-(4→5)Ara. The peak at 4.69/101.89 ppm indicates that J (H1) is associated with D (C1), indicating that the 3,4)-α-D-GalpA-(1→ residue is linked to T-Glcp via an O-1 bond, namely 3,4)-α-D-GalpA-(1→1)-Glcp. The chemical shift assignment of sugar residues in different linker sequences is shown in Table 3. The structure of WGHP-2-2 is shown in Figure 8.

### 3.5. Effect of Polysaccharide on UC Mice

#### 3.5.1. State of UC Mice

**Figure 9:**
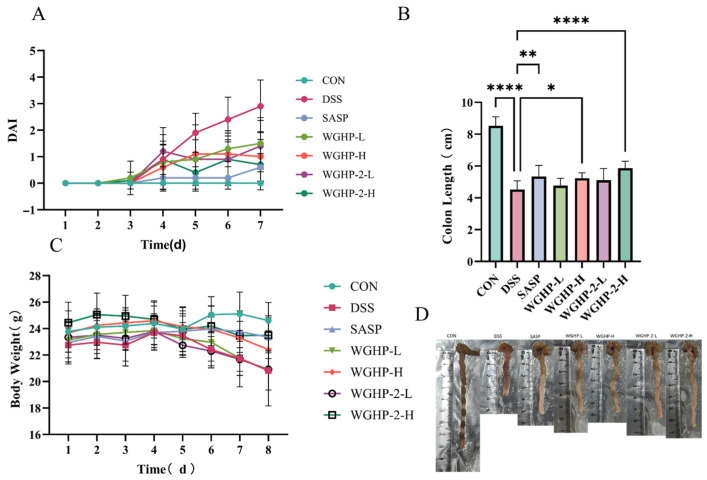
The condition of the UC mice. Note: (**A**): UC mouse DAI score; (**B**): the length of the colon in UC mice; (**C**): body weight of UC mice; (**D**): the colon of UC mice; data are expressed as mean ± SE, (*n* = 10). **** *p* < 0.0001, ** *p* < 0.01, and * *p* < 0.05.

The DAI, a widely used clinical indicator of ulcerative colitis severity, is calculated as the sum of individual scores for weight loss, stool consistency, and the presence of fecal blood. As shown in Figure 9A, the DAI remained consistently low in the CON group throughout the experiment. In contrast, mice treated with DSS exhibited a significant increase in DAI scores starting from day 4, confirming the successful induction of colitis. Treatment with either a dose of WGHP-2 or the positive control drug SASP markedly reduced DAI scores compared to the untreated DSS group, demonstrating that WGHP-2 effectively alleviates disease activity in this murine model of ulcerative colitis. These improvements were accompanied by a marked alleviation of diarrhea, rectal bleeding, and weight loss. These findings suggest that walnut husk polysaccharide WGHP-2 effectively alleviates DSS-induced UC symptoms in mice.

Figure 9C illustrates body weight changes, an indicator of overall health. In the first four days, the groups showed no significant differences in body weight. However, starting from day 5, mice in the DSS group and the low-dose WGHP-2 group exhibited varying degrees of weight loss. In contrast to the CON group, the DSS group showed a pronounced decrease in body weight by day 8. In contrast to the DSS group, mice in the SASP and WGHP-2-H groups retained body weights that were statistically similar to the controls and markedly superior to the untreated model group. This suggests that WGHP-2, particularly at higher doses, effectively mitigates DSS-induced weight loss in UC mice.

Figure 9B,D demonstrate that the mice exhibited a shortened colon compared to normal mice, indicating that colon length can serve as a measure of UC severity in mice. The CON group maintained an average colon length of 8.53 ± 0.56 cm, whereas the DSS group exhibited a marked reduction to 4.52 ± 0.55 cm, representing a significant shortening compared to controls. The average colon lengths were 5.23 ± 0.34 cm for the WGHP-H group, 4.76 ± 0.46 cm for the WGHP-L group, and 5.86 ± 0.45 cm for the WGHP-2-H group. In the case of the SASP group, the average colon length was 5.34 ± 0.70 cm, whereas the WGHP-2-L group displayed a mean length of 5.10 ± 0.73 cm. The colon shortening of WGHP-H, SASP, and WGHP-2-H groups was significantly improved. These results indicate that both WGHP and WGHP-2 alleviate colon shortening in UC mice.

#### 3.5.2. Inflammatory Profiles in Mouse Serum and Colon Tissue

**Figure 10:**
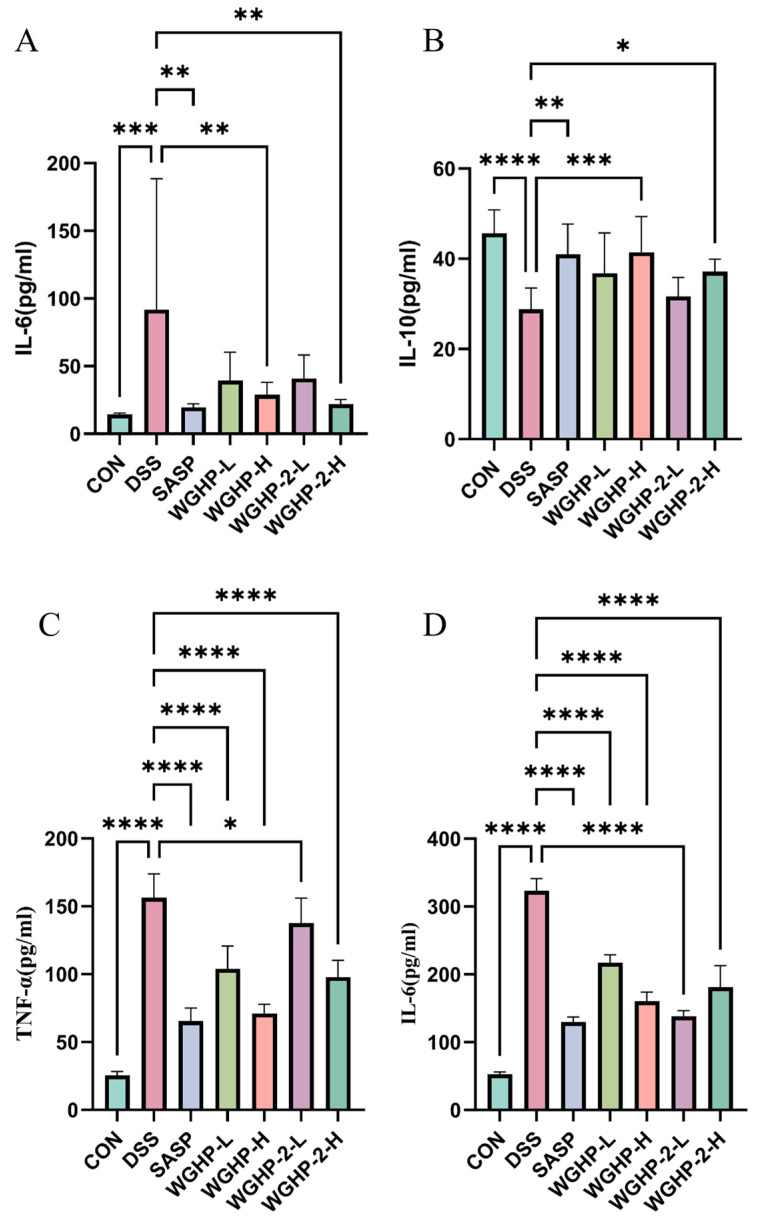
Inflammatory factors of UC mice. Note: (**A**): IL-6 content in serum of UC mice; (**B**): IL-10 content in the colon of UC mice; (**C**): TNF-α content in the colon of UC mice; (**D**): IL-6 content in the colon of UC mice. Data are expressed as mean ± SE, (*n* = 8). **** *p* < 0.0001, *** *p* < 0.001, ** *p* < 0.01, and * *p* < 0.05.

In the DSS-induced group, serum IL-6 levels were markedly elevated compared to the CON group, confirming the successful induction of a systemic inflammatory response. Notably, treatment with WGHP-2-H significantly suppressed this systemic IL-6 elevation. Both crude WGHP and its purified fraction, WGHP-2, significantly attenuated the upregulation of pro-inflammatory cytokines, specifically IL-6 and TNF-α, within colonic tissues. Conversely, both the WGHP-H and WGHP-2-H groups exhibited a significant restoration of colonic IL-10 levels compared to the DSS group. The therapeutic potential of both crude WGHP and purified WGHP-2 in mitigating UC-associated colitis is attributed to their capacity to rebalance the inflammatory milieu, characterized by the concurrent reduction in IL-6 and TNF-α and elevation in IL-10 expression. The results are shown in Figure 10.

#### 3.5.3. HE Staining Results of Mouse Colon

**Figure 11:**
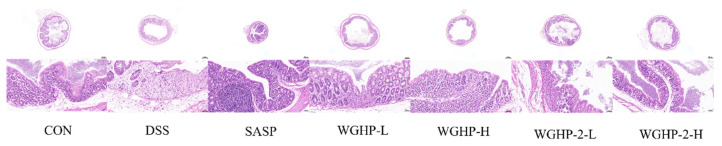
H&E staining of the colon in UC mice.

Histological examination via H&E staining (Figure 11) revealed that colonic tissues in the CON group preserved an intact mucosal architecture, with no evidence of inflammatory cell infiltration. Conversely, the DSS-treated group exhibited marked histopathological alterations, manifesting as pronounced ulceration and necrosis of the mucosal epithelium and intestinal crypts, accompanied by the profuse infiltration of inflammatory cells penetrating deep into the submucosa. The SASP (positive control) and WGHP-2-H groups demonstrated significant histological improvements compared to the DSS group. These improvements included a marked reduction in inflammatory cell infiltration and attenuation of mucosal damage. These findings indicate that WGHP-2 effectively ameliorates colonic tissue injury in UC mice.

#### 3.5.4. Immunohistochemical Analysis

Claudin-5, Occludin, and ZO-1 are essential elements of the tight junction complex. Aberrant expression and disrupted subcellular localization are closely associated with intestinal epithelial barrier dysfunction in ulcerative colitis (UC). Previous research has shown that patients with active UC exhibit significantly downregulated expression levels and markedly reduced membrane localization of these proteins. These alterations compromise the structural integrity, increase intestinal permeability, and facilitate the translocation of luminal antigens and microbial products, thereby triggering and sustaining chronic inflammatory responses.

**Figure 12:**
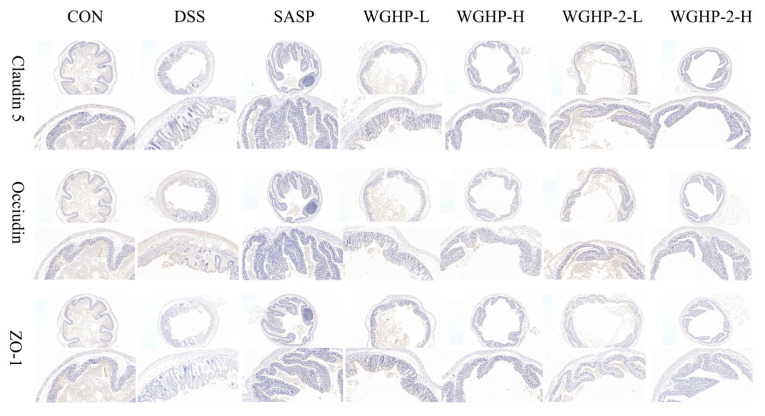
Immunohistochemical analysis of the colon in UC mice.

We assessed colonic barrier integrity in our UC mouse model by examining the expression of Claudin-5, Occludin, and ZO-1 using IHC (Figure 12). The DSS group showed a markedly decreased expression of all three TJ proteins compared to the CON group, indicating severe colonic epithelial damage and barrier dysfunction. Treatment with SASP and WGHP-2-H reinstated Claudin-5, Occludin, and ZO-1 relative to the DSS group. Notably, protein levels in the WGHP-2-H group approached those observed in the CON group. Collectively, these data indicate that high-dose WGHP-2 restores intestinal barrier homeostasis in UC mice by upregulating the abundance of key tight junction proteins.

## 4. Discussion

This study isolated and purified a homogeneous pectin polysaccharide, WGHP-2-2. HPGPC analysis revealed a molecular mass of 15.29 kDa. Monosaccharide composition analysis revealed that the polysaccharide was predominantly composed of GalA, Rha, Gal, Ara, Glc, and GlcA, with only trace amounts of GlcN detected. The molar ratio of the polysaccharide was GalA:Rha:Gal:Ara:Glc:GlcA:GlcN = 65.99:8.48:10.86:6.57:4.49:3.06:0.55. The high GalA content indicates that it is a typical pectin-type polysaccharide.

Structural characterization through methylation linkage analysis and NMR spectroscopy confirmed that WGHP-2-2 possesses a linear homogalacturonan (HG) backbone, characterized by the repetitive sequence →4)-α-D-GalpA-(1→. The →4)-α-D-GalpA-(1→2)-α-D-Rhap-(1→ sequence, representing the rhamnose galacturonic acid (RG-I) region, is integrated into the main chain. Polysaccharides represent a primary class of bioactive constituents in walnuts. A specific polysaccharide extracted from walnut green husk (WGP), with an average molecular weight of 12.77 kDa, is characterized by a backbone primarily consisting of →4)-α-D-Galp-(1→, α-D-Galp-(1→, and →2)-α-L-Rhap-(1→ linkages. This fraction has demonstrated significant efficacy in attenuating hepatic inflammation and correcting gluconeogenic dysfunction [20]. Distinctly, an acidic heteropolysaccharide isolated from walnut green husks comprises Man, Rha, GalA, Glc, Gal, Ara and Fuc. Notably enriched in GalA (53.52%), this polysaccharide alleviates immune checkpoint inhibitor-induced colitis by modulating the gut microbiota [38]. Collectively, although walnut husk polysaccharides share similar monosaccharide profiles, their divergent molar ratios and distinct glycosidic linkage patterns likely underpin their structure–activity relationships, thereby accounting for their varied biological functionalities.

In this study, WGHP-2 intervention significantly ameliorated DSS-induced colitis in a dose-dependent manner, as evidenced by attenuated body weight loss, reduced colon shortening, and diminished inflammatory cell infiltration. Mechanistically, IL-6 and TNF-α are pivotal pro-inflammatory cytokines in ulcerative colitis (UC) that compromise intestinal epithelial barrier function by activating the MLCK pathway [39,40]. Our results demonstrate that WGHP-2 not only markedly reduced the levels of these cytokines but also effectively reversed the downregulation of tight junction proteins, including ZO-1, Claudin-5, and Occludin. Specifically, the restoration of ZO-1 suggests the re-establishment of cytoskeletal anchoring complexes, while the recovery of Claudin-5 and Occludin expression indicates molecular-level repair of paracellular barrier integrity. Although direct functional permeability assays (e.g., FITC-dextran leakage test) were not performed, the concerted upregulation of these key tight junction proteins strongly implies improved intestinal barrier integrity. Given that TNF-α can induce TJ disassembly via the MLCK pathway [41], we hypothesize that WGHP-2 may disrupt the vicious cycle of inflammation–barrier damage by inhibiting TNF-α release, thereby blocking MLCK-mediated barrier disruption [42].

Ulcerative colitis is recognized as a systemic condition characterized by intestinal barrier failure, which permits microbial products to enter the circulation and potentially affect distant organs. Claudin-5 is a critical structural component of both the intestinal microvasculature and the blood–brain barrier (BBB) [43], and its expression level serves as a sensitive indicator of endothelial integrity within these barriers. By focusing on Claudin-5, our study aims to bridge the gap between local intestinal vascular damage and systemic inflammatory consequences, hypothesizing that the preservation of Claudin-5-mediated endothelial tightness is essential for preventing the spillover of inflammation from the gut into systemic circulation [44,45].

Structure–activity relationship studies have demonstrated that the immunomodulatory activity of pectic polysaccharides is highly dependent on their fine molecular structure. The existing literature indicates that the RG-I domain is the core structural motif responsible for anti-inflammatory and barrier-repairing effects, and RG-I-type pectins possessing longer and more highly branched side chains generally exhibit enhanced bioactivity [46,47]. Our prior structural characterization confirmed that WGHP-2 is a pectin-like polysaccharide rich in both HG and RG-I domains, with a primary molecular weight distribution of 15.29 kDa. Although definitive structure–activity relationships require comparative bioactivity assessments among distinct fractions or targeted chemical/enzymatic modification studies, the presence of bioactive RG-I motifs in WGHP-2, combined with its molecular weight falling within the typical range reported for active polysaccharides, provides a plausible structural basis for its therapeutic efficacy. This structural profile aligns closely with the potent anti-UC activity demonstrated in this study, suggesting that the RG-I domain likely serves as the key functional moiety responsible for WGHP-2’s therapeutic effects [48,49,50].

In this context, the therapeutic potential of high-molecular-weight polysaccharides such as WGHP-2 does not critically depend on systemic bioavailability in the conventional sense. Due to their macromolecular nature, these polysaccharides are resistant to enzymatic degradation in the upper gastrointestinal tract, enabling them to reach the colon intact—a property that is particularly advantageous for the targeted treatment of UC, a disease primarily affecting the colon [51]. Upon reaching the colon, WGHP-2 can be fermented by polysaccharide-degrading enzymes secreted by commensal gut microbiota, yielding a variety of bioactive metabolites, including SCFAs [52]. These metabolites play a central role in maintaining epithelial barrier homeostasis and suppressing inflammation. Therefore, evaluating its “local bioavailability” and microbiota-dependent biotransformation processes is more relevant to its therapeutic efficacy than monitoring systemic plasma concentrations [53].

Although this study has made preliminary progress in elucidating the structural features of WGHP and its anti-UC mechanisms, several limitations must be acknowledged: (i) Although the acute DSS-induced colitis model is widely used for initial drug screening, it does not fully recapitulate the chronic and relapsing nature of human UC. Therefore, extrapolation of the current findings to clinical applications should be approached with caution, and future validation in chronic or spontaneous colitis models is warranted. (ii) The study did not directly assess the modulatory effects of WGHP-2 on gut microbiota. There is a lack of systematic analysis of microbial community shifts and key metabolites, particularly SCFAs. While the data primarily focus on downstream endpoints related to epithelial barrier integrity and inflammation, the proposed causal chain along the microbiota–metabolite–barrier–immunity axis remains hypothetical and requires further experimental substantiation. (iii) Although changes in total protein levels of tight junction proteins were observed, critical signaling pathways—such as TLR4/NF-κB and MLCK—have not yet been thoroughly validated at the levels of protein expression or phosphorylation status using methods like Western blotting. (iv) Formal pharmacokinetic (PK), maximum tolerated dose (MTD), or sub-chronic toxicity studies were not conducted. Although no overt acute toxicity (e.g., mortality or severe weight loss) was observed during treatment—suggesting an acceptable initial safety profile—comprehensive ADME (absorption, distribution, metabolism, excretion) and toxicological evaluations remain essential prerequisites for defining the therapeutic window and advancing clinical translation.

The limitations also delineate clear and promising directions for future research. Subsequent studies will integrate metagenomics with untargeted metabolomics to deeply dissect the microbiota–host crosstalk mediated by WGHP-2. Concurrently, building upon key differentially expressed genes identified through transcriptomic analysis, we aim to systematically elucidate the underlying signaling networks at both transcriptional and post-translational modification levels—particularly phosphorylation events.

Moreover, emerging evidence suggests that certain polysaccharides can be internalized via clathrin-mediated endocytosis in the gut and subsequently enter systemic circulation [54]. This raises the intriguing possibility that WGHP-2 may exert anti-colitis effects through both local (microbiota-dependent) and systemic (host cell-directed) mechanisms—a hypothesis worthy of rigorous investigation.

Such a multidimensional, integrative omics strategy holds great promise for precisely defining the active molecular entities and therapeutic targets of WGHP-2. Ultimately, these efforts will provide a robust scientific foundation for establishing quality control standards, developing novel polysaccharide-based therapeutics, and facilitating their translation into clinical practice.

## 5. Conclusions

In conclusion, we successfully isolated and characterized WGHP-2-2, a homogeneous acidic polysaccharide from walnut green husks, and demonstrated its protective potential in a DSS-induced ulcerative colitis model. WGHP-2 intervention significantly ameliorated colitis manifestations, including attenuating weight loss, mitigating colon shortening, and preserving mucosal integrity. These protective effects were accompanied by the modulation of inflammatory mediators (IL-6, TNF-α, IL-10) and the restoration of tight junction protein expression (ZO-1, Occludin, Claudin-5), suggesting a potential role in maintaining intestinal barrier homeostasis. The abundance of RG-I domains in WGHP-2 may contribute to this bioactivity. While the current findings are limited to an acute model without microbiota or pharmacokinetic data, they provide a valuable foundation for considering walnut husk polysaccharides as functional food ingredients for intestinal health. Future research employing chronic models, multi-omics approaches, and safety evaluations is warranted to further validate its efficacy and underlying mechanisms.

## Figures and Tables

**Table 1:** Molecular weight distribution of WGHP-2 and WGHP-2-2.

Number	RT (min)	Mp (Da)	Mw (Da)	Mn (Da)
WGHP-2	35.210	37,842	47,706	31,314
	38.889	8777	10,287	7281
WGHP-2-2	36.872	13,654	15,289	9116

**Table 2:** Methylation analysis of WGHP-2-2.

Connection Method	PMAA	RT (min)	Relative Molar Ratio (%)
t-Ara*f*	1,4-di-*O*-acetyl-2,3,5-tri-*O*-methyl arabinitol	11.770	2.546
t-Rha*p*	1,5-di-*O*-acetyl-6-deoxy-2,3,4-tri-*O*-methyl rhamnitol	12.808	3.148
t-Fuc*p*	1,5-di-*O*-acetyl-6-deoxy-2,3,4-tri-*O*-methyl fucitol	13.678	1.076
1,2-Rha*p*	1,2,5-tri-*O*-acetyl-6-deoxy-3,4-di-*O*-methyl rhamnitol	15.579	5.228
t-Glc*p*	1,5-di-*O*-acetyl-2,3,4,6-tetra-*O*-methyl glucitol	16.855	1.953
t-Gal*p*	1,5-di-*O*-acetyl-2,3,4,6-tetra-*O*-methyl galactitol	17.554	3.489
t-Gal*p*A	1,5-di-*O*-acetyl-2,3,4,6-tetra-*O*-methyl galactitol	17.554	5.866
1,2,4-Rha*p*	1,2,4,5-tetra-*O*-acetyl-6-deoxy-3-*O*-methyl rhamnitol	18.475	2.325
1,2-Glc*p*A	1,2,5-tri-*O*-acetyl-3,4,6-tri-*O*-methyl glucitol	19.551	1.263
1,4-Gal*p*A	1,4,5-tri-*O*-acetyl-2,3,6-tri-*O*-methyl galactitol	19.964	54.148
1,4-Glc*p*A	1,4,5-tri-*O*-acetyl-2,3,6-tri-*O*-methyl glucitol	20.127	3.184
1,3-Gal*p*	1,3,5-tri-*O*-acetyl-2,4,6-tri-*O*-methyl galactitol	20.408	2.999
1,6-Glc*p*	1,5,6-tri-*O*-acetyl-2,3,4-tri-*O*-methyl glucitol	20.627	1.796
1,6-Gal*p*	1,5,6-tri-*O*-acetyl-2,3,4-tri-*O*-methyl galactitol	21.634	1.109
1,3,4-Gal*p*A	1,3,4,5-tetra-*O*-acetyl-2,6-di-*O*-methyl galactitol	22.066	4.600
1,2,4-Gal*p*A	1,2,4,5-tetra-*O*-acetyl-3,6-di-*O*-methyl galactitol	22.654	2.086
1,4,6-Gal*p*A	1,4,5,6-tetra-*O*-acetyl-2,3-di-*O*-methyl galactitol	23.649	1.823
1,3,6-Gal*p*	1,3,5,6-tetra-*O*-acetyl-2,4-di-*O*-methyl galactitol	24.474	1.361

**Table 3:** Summary of major glycosyl residues in WGHP-2-2 identified by methylation analysis.

Residues		H1/C1	H2/C2	H3/C3	H4/C4	H5/C5	H6/C6	-OAc
A	1,4-Gal*p*A	5.08/101.89	3.78/70.99	4.01/71.69	4.43/80.79	4.79/74.22	178.25	
B	T-Ara*f*	5.80/109.74	4.29/80.16	3.73/74.81	4.28/80.60	3.39/61.14	ns	
C	T-Rha	5.28/101.28	4.59/81.84	4.28/68.79	3.86/73.60	4.01/71.13	1.14 (1.15)/21.07	
D	1,3,4-GalpA	5.08/101.89	3.78/70.99	4.07/77.14	4.40/80.86	4.83/75.18	178.25	
E	1,3-Gal*p*	5.32/95.07	3.66/70.89	3.83/79.00	3.92/71.23	3.59/73.21	3.50/61.13	
F	T-GalpA	5.08/101.89	3.78/70.99	4.01/71.69	4.27/73.68	4.79/74.22	178.25	
G	T-Galp	3.52/70.89	3.73/67.92		3.92/71.23	3.59/73.21	3.50/61.13	
H	1,2-Rhap	5.28/101.38	4.59/81.84	4.28/68.79	3.92/73.68	4.14/71.23	1.25 (1.26)/19.48	
I	1,2,4-Rhap	5.28/101.38	4.62/81.00	4.28/68.79	3.98/80.02	4.15/70.86	1.30 (1.32)/19.72	
J	T-Glcp	4.69/103.47	3.41/72.49	3.90/73.37	3.65/72.25	3.53/73.22	3.44/63.64	
K	1,4-GlcpA	5.02/103.47	3.64/70.75	3.93/72.36	3.52/74.83	ns	178.25	
L	1,2,4-GalpA	5.08/101.89	4.20/73.37	ns	4.43/80.79	ns	178.25	
M	1,4,6-GalpA	5.08/101.89	3.78/70.99	ns	4.43/80.79	ns	172.04	2.08/22.91

Note: ns = not specified.

## Data Availability

Original materials from this work are contained in the published paper or Supplementary Materials. Direct any questions regarding these data to the corresponding author.

## Supplementary Materials

The following supporting information can be downloaded at: https://www.mdpi.com/article/10.3390/nu18091351/s1, Table S1: Measurement of inflammatory factors in UC mice; Table S2: Hydroxyl radical scavenging rate (%) of WGHP in vitro; Table S3: DPPH radical scavenging rate (%) of WGHP in vitro; Table S4: ABTS radical scavenging rate (%) of WGHP in vitro.

## Abbreviations

The following abbreviations are used in this manuscript:

UC	Ulcerative colitis
COSY	Correlated spectroscopy
HMBC	Heteronuclear multiple bond correlation spectroscopy
HSQC	Heteronuclear single quantum coherence spectroscopy
FT-IR	Fourier transform infrared spectroscopy
DSS	Dextran sulfate sodium
ZO-1	Zonula occludens-1
IHC	Immunohistochemistry
WGHP	Walnut green husk polysaccharide
