# Supplementary material for: Structural Characterization of an Acidic Polysaccharide from Walnut Green Husks and Its Therapeutic Potential in DSS-Induced Ulcerative Colitis
Source: Nutrients. 2026 Apr 24;18(9):1351. doi: 10.3390/nu18091351 (PMC13165095; doi:10.3390/nu18091351)
Supplement: Supplementary file 1 [file nutrients-18-01351-s001.zip › nutrients-4226321-supplementary.pdf]

Table S1 Measurement of inflammatory factors in UC mice

Groups	IL-6 in serum （pg/mL）	IL-6 in colon （pg/mL）	TNF-αin colon （pg/mL）	IL-10 in colon （pg/mL）
CON	2.53	47.30	22.66	45.13
CON	4.47	49.55	25.22	46.90
CON	2.88	50.67	27.52	43.36
CON	4.29	51.80	23.94	46.90
CON	4.66	54.04	29.31	45.13
CON	4.29	55.17	21.38	42.48
CON	3.93	56.29	26.75	56.64
CON	3.93	57.42	28.03	38.94
DSS	25.620	336.07	145.93	29.20
DSS	18.010	295.62	168.44	31.86
DSS	9.440	346.18	129.31	30.09
DSS	323.54	312.47	180.97	26.55
DSS	17.24	320.34	152.84	18.58
DSS	12.13	305.73	174.07	34.51
DSS	15.23	328.20	139.54	28.32
DSS	616.55	341.69	158.47	30.97
SASP	22.54	125.96	57.44	42.48
SASP	19.84	118.09	58.72	39.82
SASP	23.93	139.44	63.84	41.59
SASP	42.37	131.57	67.16	38.05
SASP	25.05	122.58	71.51	31.86
SASP	13.78	137.19	76.11	52.21
SASP	19.58	128.20	78.16	47.79
SASP	41.4	134.94	50.79	34.51
WGHP-L	63.39	220.34	79.44	36.28
WGHP-L	90.95	198.99	84.30	28.32
WGHP-L	56.41	229.33	93.76	52.21
WGHP-L	56.75	210.22	102.20	35.40
WGHP-L	54.69	224.83	110.64	24.78
WGHP-L	47.94	205.73	115.75	46.02
WGHP-L	57.44	232.70	120.36	33.63
WGHP-L	37.28	214.72	124.96	38.05
WGHP-H	60.58	159.66	60.00	49.56
WGHP-H	43.99	176.52	64.35	39.82
WGHP-H	81.18	152.92	68.18	37.17
WGHP-H	100.53	168.65	70.49	33.63
WGHP-H	48.27	140.56	72.28	46.02
WGHP-H	74.91	145.06	74.32	42.48

WGHP-H	72.72	159.66	77.90	53.10
WGHP-H	37.59	176.52	80.72	30.09
WGHP-2-L	100.92	140.56	110.38	28.32
WGHP-2-L	72.72	124.83	118.57	34.51
WGHP-2-L	63.04	146.18	124.45	25.66
WGHP-2-L	30.27	133.82	135.70	36.28
WGHP-2-L	66.59	137.19	142.86	30.97
WGHP-2-L	42.05	129.33	150.28	32.74
WGHP-2-L	81.18	148.43	155.14	27.43
WGHP-2-L	74.18	143.93	162.81	37.17
WGHP-2-H	45.95	188.88	78.41	35.40
WGHP-2-H	49.61	105.73	85.58	38.94
WGHP-2-H	36.04	196.74	92.99	39.82
WGHP-2-H	51.63	177.64	95.81	33.63
WGHP-2-H	12.59	182.13	100.15	36.28
WGHP-2-H	27.92	201.24	105.52	37.17
WGHP-2-H	36.04	192.25	110.38	41.59
WGHP-2-H	26.19	204.61	114.73	34.51

Table S2 Hydroxyl radical scavenging rate (%) of WGHP in vitro

Concentration (mg/mL)	Scavenging rate of WGHP (%)		
0.1	11.42	13.12	13.44
0.2	32.71	31.61	33.17
0.4	43.59	43.55	44.65
0.6	55.08	56.57	55.02
0.8	67.84	67.63	66.47
1	74.57	74.61	74.93
Concentration (mg/mL)	Scavenging rate of WGHP-1 (%)		
0.1	2.68	2.35	2.62
0.2	6.63	7.18	6.05
0.4	5.04	5.20	5.08
0.6	8.77	8.38	8.05
0.8	5.85	4.71	5.66
1	6.46	7.84	6.15
Concentration (mg/mL)	Scavenging rate of WGHP-2 (%)		
0.1	7.00	6.45	5.97
0.2	21.12	20.26	21.89
0.4	29.73	29.46	30.69
0.6	26.99	28.13	27.36
0.8	41.81	40.49	40.67
1	40.67	41.68	41.42
Concentration (mg/mL)	Scavenging rate of WGHP-3 (%)		

0.1	7.21	7.50	7.79
0.2	10.12	10.43	11.00
0.4	22.39	20.65	20.71
0.6	29.90	29.59	28.94
0.8	46.24	46.48	47.80
1	47.14	47.01	48.61

Table S3 DPPH radical scavenging rate (%) of WGHP in vitro

Concentration (mg/mL)	Scavenging rate of WGHP (%)		
0.2	5.56	5.05	5.27
0.4	15.49	16.07	15.74
0.6	28.32	29.19	27.71
0.8	37.34	36.87	36.57
1	68.94	70.26	70.15
Concentration (mg/mL)	Scavenging rate of WGHP-1 (%)		
0.2	8.49	9.18	9.31
0.4	13.02	13.62	12.75
0.6	13.87	13.35	14.01
0.8	16.36	14.80	15.41
1	22.09	21.02	22.10
Concentration (mg/mL)	Scavenging rate of WGHP-2 (%)		
0.2	2.28	3.08	2.80
0.4	10.19	9.25	9.53
0.6	11.48	10.22	10.51
0.8	16.32	17.01	16.30
1	31.96	32.45	32.48
Concentration (mg/mL)	Scavenging rate of WGHP-3 (%)		
0.2	26.25	24.90	25.26
0.4	26.45	26.44	25.46
0.6	32.94	35.45	37.23
0.8	25.28	25.88	24.82
1	22.74	24.42	24.31

Table S4 ABTS radical scavenging rate (%) of WGHP in vitro

Concentration (mg/mL)	Scavenging rate of WGHP (%)		
0.1	26.13	25.78	25.45
0.2	30.32	29.86	29.59
0.3	38.17	37.59	37.69
0.4	69.81	69.19	69.93
0.5	82.04	83.32	80.45
Concentration (mg/mL)	Scavenging rate of WGHP-1 (%)		

0.1	12.31	10.73	11.05
0.2	12.77	12.98	11.88
0.3	16.99	17.13	16.99
0.4	24.71	23.39	23.69
0.5	27.09	26.80	25.83
Concentration (mg/mL)	Scavenging rate of WGHP-2 (%)		
0.1	21.61	20.17	20.47
0.2	33.94	33.94	34.27
0.3	48.12	48.79	49.12
0.4	71.35	71.70	71.66
0.5	91.79	90.36	91.99
Concentration (mg/mL)	Scavenging rate of WGHP-3 (%)		
0.1	13.68	13.86	13.45
0.2	16.80	16.80	16.61
0.3	18.71	19.57	19.08
0.4	23.45	23.80	23.98
0.5	25.18	25.14	25.85
